# Self-Rated Health and Public Health: A Critical Perspective

**DOI:** 10.3389/fpubh.2013.00015

**Published:** 2013-05-20

**Authors:** Andrea E. Bombak

**Affiliations:** ^1^Department of Community Health Sciences, University of ManitobaWinnipeg, MB, Canada

## Introduction

Self-rated health (SRH) is a simple, easy to administer measure of general health. It is a valid and reliable measure among those without cognitive impairment. Initially, it replaced clinical assessments in survey research (1). It is commonly used in psychological research, clinical settings, and in general population surveys. SRH is typically measured as a single-item, the most common wording of which is “In general, would you say your health is” with the response items “excellent,” “very good,” “good,” “fair,” or “poor.” Early studies using SRH involved assessing the relationship between SRH with sociodemographic, physical health, and psychosocial variables (e.g., 2, 3). Additional uses of SRH involved investigating relationships between health constructs, sociodemographic, physical, and psychosocial variables, clarifying measurement issues, attempting to explain health and illness behavior, or describing populations’ health (e.g., 2–4). SRH was found to be at least moderately associated with physicians’ assessments of health (2, 4–8). SRH allows respondents to prioritize and evaluate different aspects of their health, maximizing the measure’s sensitivity to respondent views of health (9). SRH’s somewhat abstract nature also affords researchers the opportunity to examine the cognitive processes involved in evaluating self-health (9). A significant, independent effect of SRH on mortality has been demonstrated in numerous studies and diverse populations (10–12). Other health outcomes including chronic disease incidence, diabetes complications, physical and cognitive functional limitations, health services use, and clinical biomarkers have also been investigated (13–20). A large body of literature concerning SRH, its determinants, and its outcomes has accumulated from studies conducted throughout the world.

Recently, public health researchers have begun to deploy SRH either implicitly or explicitly to gage individuals’ willingness to engage in behavior modification. This paper critically assesses the literature on SRH and health behaviors. It examines the theoretical and ethical utility of SRH as a tool in health-related behavior modification.

## SRH and Health Behaviors

A complex relationship exists between SRH and health-related behaviors. Health-related behaviors included in SRH studies often include smoking status, dietary assessments, physical activity, body mass index (BMI) or presence of obesity, and alcohol activity (21). Often these health behaviors are included as covariates, rather than explanatory or outcome variables (e.g., 18, 21, 22). Likewise, health-related behaviors have been used as control variables in studies exploring SRH and mortality. Health behaviors have been shown to mediate the relationship between SRH and mortality, and this effect often differs by gender and/or duration of effect (15, 23, 24). Other studies, however, have only seen a fairly weak mediating influence of health behaviors on SRH and mortality (25–27). Some studies have found only weak or irrelevant associations with SRH (28–30). Conflicting findings have emerged concerning SRH and all of smoking, alcohol consumption, and dietary behaviors (17, 20, 31–43). Layes et al. (44) determined that individuals who engaged in healthy lifestyles were actually more likely to be pessimist regarding their health statuses.

Manderbacka (45) found some Finnish adults clearly have adopted health messaging and incorporated risk behaviors and fitness-centric responses in their self-evaluations. Krause and Jay (46) found younger and better educated Americans were more likely to incorporate health behaviors in their assessments of self-health, and similar age-related findings were found among Australian women (47). These findings reflect the groups in which health messaging may have had the highest rates of absorption. The reviewed studies provide some evidence that health behaviors affect SRH. However, the relationship is ambiguous, not always in expected directions, and mediated by age, gender, and ethnicity. Such studies also do not consider the manner through which some health behaviors, while ostensibly health-damaging, may serve to promote pleasure and stress-relief, and thus may be mental-health enhancing.

## SRH and Behavior Modification

In reviewing the literature on SRH and health behaviors, it is evident that some studies adopt a corrective attitude relating to SRH. They seek to determine the congruence of an individual’s assessment of health with an objective rating of health based on medical diagnoses or health behaviors. These studies appear not to be designed to better understand SRH but rather to use it to assess individuals’ level of “awareness” regarding their health status. It is assumed that those who rate their health favorably, in spite of objective indications of poor health, must have their SRH corrected through education and the adoption of appropriate behavior changes.

For example, researchers have adopted a critical tone in studies in which respondents have reported generally favorable health while suffering from chronic conditions such as diabetes, hypertension, and obesity and adopting risky health behaviors such as smoking, inactivity, lack of produce consumption, or soda consumption among Australian Aboriginal, and American Appalachian, Black, Hispanic, and Latino populations (48–51). For example, Griffith and colleagues refer to the distortion they believe is evident in Appalachians’ SRH. Appalachia residents rate their health more highly than their health behaviors and objective health status might suggest. This is viewed as an impediment to initiating behavior change, and thus the authors recommend that “Education, therefore, must be targeted at promoting appropriate views of health and the need for improved health…formation and delivery of new public messages and programs for rural Appalachians should be focused on people who are unhealthy and have poor health behaviors, but believe they are healthy” (2011, 7 of 8). That is, because respondents’ SRH was not associated with objective health measures, it is “inaccurate” and must be corrected. Participants will then be willing to acknowledge their “actual” poor health and engage in appropriate behavior change.

Interestingly, Kepka et al. (48) acknowledges that SRH is positively associated with health beneficial behaviors in recent Latino immigrants, although more frequent engagement in health behaviors is deemed necessary. Despite its relationship to behavioral factors, the authors are concerned that lower SRH does not predict higher BMI. The authors are further troubled that respondents reported improvements or stability to their quality of life and health following immigration, despite weight gain. This suggests a particularly narrow definition of health. As stated by the authors, “health education strategies should be targeted at recent Latino immigrants…to heighten awareness regarding overweight and obesity risk and help individuals more accurately link perceptions of one’s health and BMI” (48, p. 541). Similarly, Burroughs et al. (49) suggest that the optimist SRH reported by overweight African Americans may be the result of lower levels of weight dissatisfaction and greater social acceptance of excess weight. This degree of self-acceptance is considered dangerous to health by the authors. They suggest it has important practice implications “as individuals who do not perceive that they are overweight are unlikely to take action to control or lose weight” (49, p. 1404).

The underlying assumption of the reported implications of these studies is the need for education concerning individuals’ own perceptions of health. It is proposed that the overly health-optimist individuals must be educated regarding their health status in order to ensure that they more accurately assess their health. It is presumed that this “accurate” evaluation of poor health status will motivate individuals to engage in behavioral modification, despite, for example the Latino immigrants’ association between at least some degree of healthful behavior and SRH (48). Self-acceptance or resilience regarding health when faced by disease or socioeconomic difficulties appears to not be considered a positive coping method. Other, holistic determinants of health, to which individuals may refer when rating health, are not considered. Instead, researchers focus on the need for individuals to take responsibility for conceptualizes of health held by the researchers, and to initiate changes to meet these external ideals.

## Implications of Deploying SRH in Behavior Modification

Self-rated health is inherently a subjective measure of internal perceptions and priorities. Thus, the use of SRH to rectify individuals’ impressions of their health, in order to motivate behavior change, suggests a problematically non-salutogenic, individualistic, and moralist perspective. This utilization of SRH has important ethical and practice implications.

In their review on SRH and mortality, Idler and Benyamini (11) speculated that one of the dimensions of SRH that may be inaccessible to researchers was individuals’ knowledge of health-related behaviors and adherence. Researchers have subsequently used this potential relationship to lend SRH studies clinical and applied significance. This presumes that a greater understanding of SRH will aid in the development of more effective behavioral interventions. In turn, these expectations are founded on the assumption that individuals with less-than-optimal health must first be made to recognize their problematic health status, acknowledge their need for health behavior modification, and then choose to undertake more salubrious lifestyle choices.

Within this context, SRH is no longer used to explore an individual’s unique perceptions of their health or as a proxy for clinical health. Instead, individuals will be expected to rate their health according to others’ standards, identify deficiencies, and then correct their behaviors to achieve “better” health. As individuals may incorporate numerous factors in their self-perceptions of health, this assumes individuals have control over exceptionally comprehensive conceptualizations of health. Significantly, health has taken on a great deal of moral weight. Labeling someone “unhealthy” has significant social ramifications, and it is imperative researchers be respectful of the daily contexts of individuals’ choices and their unique perspectives on wellbeing.

Of importance is that no simple relationship exists between SRH and lifestyle choices. Rather, SRH and behavior may be mediated by sociodemographic or cultural factors, and more positive SRH may not seemingly be produced by seemingly “health-conducive” behaviors. To assess willingness to engage in behavior modification, a more direct measure regarding individuals’ lifestyle health behavior modification intentions may be necessary. Furthermore, SRH researchers often employ a limited, biomedical definition of “health,” which ignores the multi-faceted nature of SRH. Perhaps unintentionally, they also seek to “correct” the perspective of the populations they are studying. This is particularly problematic in studies conducted in disadvantaged or minority populations, wherein cultural discrepancies between researchers and participants in definitions of health may exist. The validity and value of SRH has frequently been related to its established relationship with mortality, which is often found to be independent of clinical or physician assessments and to surpass these measures in predictive power. Clearly, individuals are capable of recognizing their own state of wellbeing, regardless of whether this reflects the views held by practitioners and researchers, and they may rely on dimensions of health inaccessible to researchers (2, 52). Of paramount importance, researchers must consider whether it is ethical to convince individuals that their health is poorer than they perceive, if this affects mortality and morbidity risk.

Risky health behaviors may relieve stress. As such, they may be “health”-motivated in a particular sociocultural context. They are not necessarily actions borne of ignorance. While incongruent with objective measures of health, some positive ratings may be demonstrative of resilience. It is these complex inquiries that are too often unexplored in preventive health, and these are the fascinating and essential avenues of research available to future investigators of SRH.
